# Spike train entropy and information transmission for a mathematical model of a bursting neuron

**DOI:** 10.1007/s10827-026-00931-y

**Published:** 2026-04-18

**Authors:** Peter K. D. Hovland, Alexandria Kissas, Elisa H. Welch, John T. Birmingham

**Affiliations:** Department of Physics and Engineering Physics, Santa Clara University, 500 El Camino Real, Santa Clara, CA 95053 USA

**Keywords:** Bursting, entropy, information, model neuron

## Abstract

In this paper we present calculations of the entropy and information transmission associated with spike trains produced by the circle/circle bursting model neuron in response to filtered white-noise stimuli. For most computations, we treated the bursts as unitary objects and estimated the entropy from the time intervals between the first spikes in consecutive bursts. In one case, we considered the intervals associated with all the spikes in the burst train or only the first and last spikes of each burst. We found that the entropy per burst was maximized when the stimulus was well matched to the neuron’s natural burst frequency and that the entropy/spike increased considerably when the duration of the burst was considered. Moreover, for a noisy stimulus for which the deterministic part of the stimulus was close to the natural burst frequency but most of the noise was at much higher frequencies, the bursting neuron transmitted considerably more information in bits/spike than a spiking model neuron constructed using similar ionic conductances.

## Introduction

Most messages from neurons to their targets are mediated by propagating voltage pulses known as action potentials or spikes. Spikes are largely stereotyped, and so it is their number and/or timing that carry the neural signal. Some neurons are capable of “bursting”; they produce a series of spikes in rapid succession followed by a period of quiescence until the next burst is generated. Bursts can result from the intrinsic properties of a single neuron, or they can be a network phenomenon. Functionally, they have great importance in many vertebrate and invertebrate nervous systems (Zeldenrust et al., 2018 and references therein). Bursts are essential for rhythmic motor pattern generation (Marder & Calabrese, 1996) and synchronization (Belykh et al., 2005; Izhikevich, 2000; Rulkov, 2001). They are more reliable than single spikes and can overcome synaptic transmission failure, facilitate transmitter release, and evoke long-term potentiation at synapses (Lisman, 1997).

The amount of variability in a train of spikes can be quantified by calculating its entropy, which can be reported in bits/s or bits/spike. A larger entropy corresponds to more variability in the spike patterns that can be produced, and, hence, there is a greater potential for the neuron to describe any particular stimulus in a spike train. Spike train entropy depends, not just on the rate of spiking, but also on the manner in which spikes are produced. For example, for a given average spike rate, the entropy is larger when the spikes are generated independently and unpredictably than when previous spikes influence the timing of those that follow. How much a neuron encodes about a stimulus in a spike train can be quantified using information theory (Shannon, 1948). The information transmitted is the total entropy (the “information capacity”) minus the “noise entropy” associated with spike trains produced when a specific stimulus is repeatedly presented (Strong et al., 1998). While a large spike train entropy potentially translates into a large information, this occurs only if the variability in spike trains correlates with features in the stimulus, rather than being random or due to other factors.

All else being equal, one would expect that the entropy/spike associated with spike trains produced by a bursting neuron should be considerably smaller than for a spiking (i.e. non-bursting) neuron firing at the same rate: the timing of spikes within a burst is highly stereotyped; once a burst starts, the other spikes follow in a relatively predictable fashion and are, in a sense, a redundant message. How much does variation in the timing of spikes within bursts contribute to the entropy? If, in addition, there are correlations in the timing of consecutive bursts, how does this affect the entropy? Sensory coding and information transmission by bursting neurons have been studied in a few diverse experimental and computational systems, as will be discussed later in the text. The answer to the first question is that the timing of individual spikes within a burst can be important, but that often consideration of only coarse features (e.g. the burst duration or the number of spikes in a burst) is sufficient. The second question has not previously been addressed.

In this paper, we describe a computational investigation of entropy (mainly) and information made to try to provide answers to these questions for a particular model neuron. The model used was the single-compartment, endogenously-bursting, circle/circle neuron, and the stimulus was white noise, low-pass filtered at frequencies between 0.1 and 1000 Hz. We analyzed spike trains in which the timing of consecutive bursts was largely independent and those in which it was correlated over many cycles. While we mainly treated the bursts as unitary objects, in one set of computations we calculated the contributions to entropy from the spikes within bursts.

Our results complement and extend those of previous studies (Eyherabide et al. 2008, 2009; Kepecs and Lisman 2003, 2004; Oswald et al. 2007; Reich et al. 2000; P. Reinagel et al. 1999; Schlungbaum et al. 2025). There are three main findings of our investigation. First, when considering bursts as unitary objects, spike train entropy was maximized when the stimulus varied on a time scale that was comparable to the neuron’s natural burst frequency. Second, entropy/spike for a burst train increased considerably when both the first and last spike of each burst were considered. Third, and most interestingly, for a certain well-chosen stimulus, the bursting neuron was able to transmit considerably more information/spike than did a spiking neuron that contained similar ionic conductances, even when treating the bursts as unitary objects and ignoring their structure. This was the case when the deterministic part of the stimulus was well matched to the natural bursting frequency and most of the noise was well above this frequency and filtered out by the neuron’s slow dynamics.

## Methods

### Mathematical model

Our interest in encoding by bursting neurons originated in experimental studies (Birmingham et al., 1999) of the gastropyloric receptor neuron 2 (GPR2), a mechanoreceptor in the stomatogastric nervous system (Harris-Warrick et al., 1992) of the crab *Cancer borealis*, that has both a spiking and an endogenously-bursting mode (Birmingham et al., 1999; Katz et al., 1989). When the neuron is in the bursting mode, we found that the instantaneous GPR2 spike rate (defined as the inverse of the interspike interval) during a burst was often approximately parabolic (unpublished observation), a characteristic of the circle/circle model (Izhikevich, 2006). This model, which well describes activity of the intrinsically-bursting R15 neuron in *Aplysia* (Arvanitaki & Chalazonitis, 1955; Tauc, 1954), contains four non-inactivating time- and voltage-dependent conductances. Instantaneous sodium and fast potassium currents plus a leak current produce voltage spikes, and the addition of decoupled slow sodium and potassium currents results in bursting. In response to an injected current (density) *I*(*t*), the membrane potential *V*(*t*) is governed by the following equations:

1 $$\begin{aligned}&C\dot{V}=I\left(t\right)-{g}_{\mathrm{L}}\left(V-{E}_{L}\right)-{\stackrel{-}{g}}_{\mathrm{N}\mathrm{a}}{m}_{\infty}\left(V\right)\left(V-{E}_{Na}\right)-{\stackrel{-}{g}}_{\mathrm{K}}n\left(V-{E}_{L}\right)\\&-{\stackrel{-}{g}}_{\mathrm{N}\mathrm{a},\mathrm{s}\mathrm{l}\mathrm{o}\mathrm{w}}{m}_{\mathrm{s}\mathrm{l}\mathrm{o}\mathrm{w}}\left(V-{E}_{Na}\right)-{\stackrel{-}{g}}_{\mathrm{K}\left(\mathrm{M}\right)}{n}_{\mathrm{s}\mathrm{l}\mathrm{o}\mathrm{w}}\left(V-{E}_{K}\right)\end{aligned}$$

2 $$\dot{n}=\left({n}_{\infty}\left(V\right)-n\right)/{\tau}_{n}$$

3 $${\dot{m}}_{\mathrm{s}\mathrm{l}\mathrm{o}\mathrm{w}}=\left({m}_{\mathrm{s}\mathrm{l}\mathrm{o}\mathrm{w},{\infty}}\left(V\right)-{m}_{\mathrm{s}\mathrm{l}\mathrm{o}\mathrm{w}}\right)/{\tau}_{\mathrm{N}\mathrm{a},\mathrm{s}\mathrm{l}\mathrm{o}\mathrm{w}}$$

4 $${\dot{n}}_{\mathrm{s}\mathrm{l}\mathrm{o}\mathrm{w}}=\left({n}_{\mathrm{s}\mathrm{l}\mathrm{o}\mathrm{w},{\infty}}\left(V\right)-{n}_{\mathrm{s}\mathrm{l}\mathrm{o}\mathrm{w}}\right)/{\tau}_{\mathrm{K}\left(\mathrm{M}\right)}$$

5 $${x}_{\infty}={\left(1+exp\left\{\left({V}_{1/2}-V\right)/k\right\}\right)}^{-1}$$

where *x* = *m*, *n*, *m*_slow_, *n*_slow_ are gating variables.

Numerical values (below) for membrane properties identical to those of Izhikevich (2006) were used with one exception—the maximal conductance of the slow sodium current ${\stackrel{-}{g}}_{Na,slow}$ was reduced from 3 to 2 mS/cm^2^:

$$\begin{array}{ll}C=1&\mu F/{cm}^2\\\\g_L=8,{\overset-g}_{Na}=20,{\overset-g}_K=10,{\overset-g}_{Na,slow}=2,{\overset-g}_{K(M)}=20&mS/{cm}^2\\\\E_L=-80,\;E_{Na}=60,E_K=-90,m_\infty:V_{1/2}=-20,\;k=15,n_\infty:V_{1/2}=&{}\\-20,k=15,m_{slow,\infty}:V_{1/2}=-40,k=5,n_{slow,\infty}:V_{1/2}=-20,k=5&mV\\\\\tau_n=1,\tau_{Na,slow}=20,\tau_{K(M)}=40&ms\end{array}$$

We chose to decrease ${\stackrel{-}{g}}_{Na,slow}$ to reduce the average number of spikes per burst to facilitate calculations of the contribution of intraburst properties to the entropy (Fig. 6B). We also worked with a version of the model in which ${\stackrel{-}{g}}_{\mathrm{N}\mathrm{a},\mathrm{s}\mathrm{l}\mathrm{o}\mathrm{w}}$ was set to zero and ${\stackrel{-}{g}}_{\mathrm{K}\left(\mathrm{M}\right)}$ was reduced to 4.5 mS/cm^2^. In this case, the neuron remained in a spiking mode and had a spike rate that was very close to that of the bursting neuron for a constant 9 µA/cm^2^ stimulus. In all simulations, we used Euler’s method with a time step of 25 µs to integrate Eqs. 1–4 numerically. Spike times were defined as the times a particular voltage (-20 mV) was exceeded.

### Entropy and information calculations

In our calculations of spike train entropy, we applied a combination of techniques. The “direct method” (Strong et al., 1998), which assigns a 0 or 1 to each bin and then considers the probability distribution of increasingly long binary words, is a standard approach. However, because our bin size (1 ms) was small compared to the average time between bursts (> 100 ms), and because the timing of consecutive bursts was often correlated, using this technique to calculate entropy was challenging because of word length. We will return to this point later in this section. Instead, we applied the technique of Rieke et al. (1993), in which maximum-likelihood estimates of the entropy are calculated from the probability distributions of interspike intervals (ISIs). In our analysis of spike trains consisting of bursts, we modified the approach to consider only certain spikes (and the times between them) when computing the entropy. For most computations, we treated the bursts as unitary objects and estimated the entropy from the intervals between the first spikes in consecutive bursts (“periods”). For one set of calculations, we considered all the spikes in the burst train, as well as just the first and last spikes of each burst. In the latter case, the intervals varied between longer interburst intervals (IBIs, the times between the last spike in a burst and the first spike in the following burst) and shorter burst durations (the times between the first and last spike in a burst). The general approach to estimating entropy using time intervals is summarized below.

Consider an experiment in which time intervals *τ*_1_, *τ*_2_, …, *τ*_*N*_ have been calculated from spike times *t*_1_, …, *t*_*N*_ (*τ*_1_ = *t*_1_ , *τ*_*n*_ = *t*_*n*_ – *t*_*n−*1_ for *n* > 1) and rounded into bins with width Δ*t*. We took Δ*t* to be 1 ms, comparable to the width of an action potential. An asymptotically decreasing sequence of upper bounds on the entropy associated with the sequence of intervals can be calculated by considering the probabilities of the intervals themselves, of consecutive pairs of intervals, of triples, and so on (Rieke et al., 1993). The first three upper bounds to the entropy per spike for resolution Δ*t* are

6 $${S}_{0}=-\sum_{{\tau}_{n}}P\left({\tau}_{n}\right){\mathrm{l}\mathrm{o}\mathrm{g}}_{2}P\left({\tau}_{n}\right)$$

7 $${S}_{1}=-{\sum}_{{\tau}_{n},{\tau}_{n-1}}P\left({\tau}_{n},{\tau}_{n-1}\right){\mathrm{l}\mathrm{o}\mathrm{g}}_{2}P\left({\tau}_{n}|{}_{n-1}\right)$$

8 $${S}_{2}=-{\sum}_{{\tau}_{n},{\tau}_{n-1},{\tau}_{n-2}}{P\left({\tau}_{n},{\tau}_{n-1},{\tau}_{n-2}\right)\mathrm{l}\mathrm{o}\mathrm{g}}_{2}P\left({\tau}_{n}|{\tau}_{n-1},{\tau}_{n-2}\right)$$

where, for example, $P\left({\tau}_{n},{\tau}_{n-1},{\tau}_{n-2}\right)$ is the probability of three particular intervals occurring in order and $P\left({\tau}_{n}|{\tau}_{n-1},{}_{n-2}\right)$ is the conditional probability of the third interval given the previous two. The expressions for higher-order upper bounds can be written in a similar fashion. The *S*_*i*_ form a monotonically decreasing sequence of upper bounds on the entropy. When the order *i* of the calculation is sufficiently large such that $\tau{}_{n}$ and ${\tau}_{n-i}$ are uncorrelated, the expression for ${S}_{i}$ reduces to that for ${S}_{i-1}$, and all higher-dimensional upper bounds converge to ${S}_{i-1}$, which is an accurate estimate of the entropy. For example, if spiking is described by a homogeneous Poisson process and the timing of each spike is independent of previous ones, the entropy is *S*_0_.

Whether there are correlations or not, trustworthy calculations of *S*_*i*_ necessitate accurate probability distributions and, in general, require more data as *i* increases. To correct for the finite size of a data set, we applied a technique introduced by Strong et al. (1998) and extended by Dorval (2008). We plotted estimates of *S*_*i*_ made using fractions $\left(\frac{1}{5},\frac{1}{4},\frac{1}{3},\frac{1}{2},1\right)$of the data set versus the data fraction reciprocal (5, 4, 3, 2, 1) and fit the points to a quadratic function. In practice, rather than dividing a single spike train into fractions, we made multiple trials (e.g. 60) and considered all intervals in fractions of the trials (e.g. 12, 15, 20, 30, 60). The zero crossing of the fitted curve corresponds to a value of *S*_*i*_ for an infinitely large data set (Strong et al., 1998). If the estimate using the entire data set was no more than 2% smaller than the extrapolated value for an infinite data set, we took the latter as the value of *S*_*i*_; if the difference was more than 2%, we rejected the calculation altogether (Dorval et al., 2008). In cases for which we had sufficient data to calculate upper bounds accurately, but the upper bounds themselves continued to decrease because of long-range correlations, we plotted *S*_*i*_ versus $\frac{1}{i+1}$, the reciprocal of the “dimension” *i* + 1 associated with *S*_*i*_. The intercept of a linear fit with the vertical axis, *S*_*inf*_, was taken to be the estimate of the entropy when considering infinite correlations (Dorval, 2008; Dorval et al., 2008). These linear fits were better in some cases than in others, and including different sets of points in the fit led to uncertainty in *S*_*inf*_ in some cases, as we discuss later in the text (Fig. 4B). As a consistency check, we used both the direct method (Strong et al., 1998) and the interval approach described above to calculate the entropy of burst trains produced in response to a white-noise stimulus low-pass filtered at 10 Hz. Only the start times and hence the periods were considered in this calculation. In Sect. 3.2 we show that *S*_0_ is a good measure of the entropy; the start times of consecutive bursts were uncorrelated. Using the interval method, *S*_0_ was computed to within 0.5% accuracy using 100 s of simulated data. The direct method failed using 1000 s of data. With 10,000 s of data using the direct method, extrapolation to infinite word length using words between 100 and 166 bits long yielded an entropy that agreed with *S*_0_ within 2%.

The information carried by a spike train is the difference between the total entropy and the noise entropy (Strong et al., 1998). The total entropy *S*_*tot*_ associated with the entire stimulus ensemble was calculated from a long stimulus as described above. To compute the noise entropy, we presented the neuron with repetitions of a short stimulus that was the sum of the same repeated waveform and a random one that varied trial to trial. The intervals from all trials were pooled together, *S*_*noise*_ was determined using the approach described above, and the information was calculated as *I* = *S*_*tot*_ - *S*_*noise*_. The corresponding coding efficiency can be defined as *I* / *S*_*tot*_. As we discuss in Sect.  3.4, we initially chose to use white noise low-pass filtered at 3 Hz for the repeated waveforms and white noise low-pass filtered at 1000 Hz for the random waveforms and afterwards considered other combinations of the signal and noise frequencies. A temporal resolution of 1 ms was the smallest for which calculations were possible in the computation time allotted. This could have resulted in a slight underestimation of the information (Reinagel & Reid, 2000; Rokem et al., 2006).

We should add a few comments about the noise entropy calculations. First, in experimental investigations, a stimulus is typically chosen to have a physiologically-relevant duration. In this work, a 1-s duration stimulus was an arbitrary but practical choice. A stimulus much longer than 1 s would have resulted in prohibitively long calculations.

Second, the random waveforms used were chosen to be of the same general form as the repeated waveforms—white noise up to a sharp cutoff frequency (either 100 or 1000 Hz). A biologically-inspired choice would have had a more complicated dependence on frequency (Diba et al., 2004; Faisal et al., 2008; Manwani & Koch, 2001; Stevens, 1972). While Johnson noise associated with thermal agitation of charge carriers is white, it is several orders of magnitude smaller than the current noise associated with fluctuations related to the number of open channels. We would expect the power spectrum of the latter to drop off approximately as *f*^− 1^ beginning at frequencies well below 100 Hz or, when considering only a single channel type, be Lorentzian and start out constant but eventually drop off as *f*^− 2^ beyond a critical frequency. All that said, for a fast sodium channel, this transition would occur well above 100 Hz.

Third, given spike times *t*_1_, …, *t*_*N*_ in a trial, we calculated *τ*_*n*_ = *t*_*n*_ – *t*_*n−*1_ only for *n* > 1, i.e. there was one fewer interval than spike that was considered. We did this because there was no reasonable way to find the one more interval and keep the duration of a trial fixed. (Unlike in the case of a Poisson process, *τ*_1_ = *t*_1_ is not valid.) So, while we report entropy (in bits) per burst or spike, in fact, we calculated entropy per interval. A calculation of *S*_*tot*_ involved thousands or even millions of bursts, and so the discrepancy was insignificant. For the shorter trials used to calculate *S*_*noise*_, in which there were typically nine or so bursts, there was a slight difference.

Fourth and last, for the short trials used to generate *S*_*noise*_ for the bursting neuron, we found that entropy was slightly underestimated unless we waited some time before making our 1-s measurements. The bursts, trial to trial, started out a bit too synchronized (compared to later on) even in the presence of considerable noise, and so we needed to “toss” enough of the beginning of each trial such that this was no longer the case. Because the time required for these calculations scaled approximately linearly with the total duration of an experiment, we first determined the minimum time that had to be discarded before doing the much longer computations required to obtain the noise entropy. Empirically, we found that tossing the first 1.5 s of data (and using the next 1 s) was indistinguishable from tossing the first 4 s (and using the next 1 s). More specifically, we calculated *S*_0_ and *S*_1_ when tossing 0, 1.5 and 4 s, 40 times each. For each calculation, we used a different repeated waveform and made 6000 trials. The average *S*_0_ calculated was 4.70, 4.84, and 4.82 bits/burst, respectively. A one-way ANOVA showed that *S*_0_ depended significantly (*p* = 0.0125) on the time tossed. Using a post-hoc Tukey HSD test, we found that value of *S*_0_ when tossing 0 s was significantly different than when tossing 1.5 s (*p* = 0.0179) or 4 s (*p* = 0.0448) but that the results for *S*_0_ under the last two conditions were not significantly different (*p* = 0.935). Similarly, the average *S*_1_ calculated was 4.10, 4.27, and 4.28 bits/burst, respectively. A one-way ANOVA showed that *S*_1_ depended significantly (*p* < 10^− 4^) on the time tossed, and significant differences were found between tossing 0 s and 1.5 s, and 0 s and 4 s, but not 1.5 s and 4 s (*p* = 0.00077, 0.00028, 0.959, respectively, Tukey test).

When bursts are generated independently and the entropy associated with the start times of bursts is well approximated by *S*_0_, there is a less computationally expensive approach than considering all ISIs for calculating the contributions of the intraburst spike timing to the entropy. Let *b*_*n*_ generically describe spike timing within the *n*^th^ burst, which itself follows the *n*^th^ period τ_*n*_. For a particular temporal resolution Δ*t* the entropy is

9 $$S=-\sum_{{\tau}_{n},{b}_{n}}P\left({\tau}_{n},{b}_{n}\right){\mathrm{l}\mathrm{o}\mathrm{g}}_{2}P\left({\tau}_{n},{b}_{n}\right)$$

We described the variability of the bursts *b*_*n*_ in two ways: (1) by simply finding the probabilities for the various number of spikes (or, equivalently intraburst ISIs) in a burst, and (2) by finding the probabilities of all sequences of intraburst ISIs, rounded to resolution Δ*t*. In both cases, if spike timing within bursts is independent of the time since the start of the last burst, $P\left({\tau}_{n},{b}_{n}\right)=P\left({\tau}_{n}\right)P\left({b}_{n}\right)$, and Eq. 9 simplifies to

10 $$S=-\sum_{{\tau}_{n}}P\left({\tau}_{n}\right){\mathrm{l}\mathrm{o}\mathrm{g}}_{2}P\left({\tau}_{n}\right)-\sum_{{b}_{n}}P\left({b}_{n}\right){\mathrm{l}\mathrm{o}\mathrm{g}}_{2}P\left({b}_{n}\right)$$

Here the total entropy is the sum of the contribution due to the variability in the periods plus that due to the variability of the bursts themselves.

### Computations

**Fig. 1:**
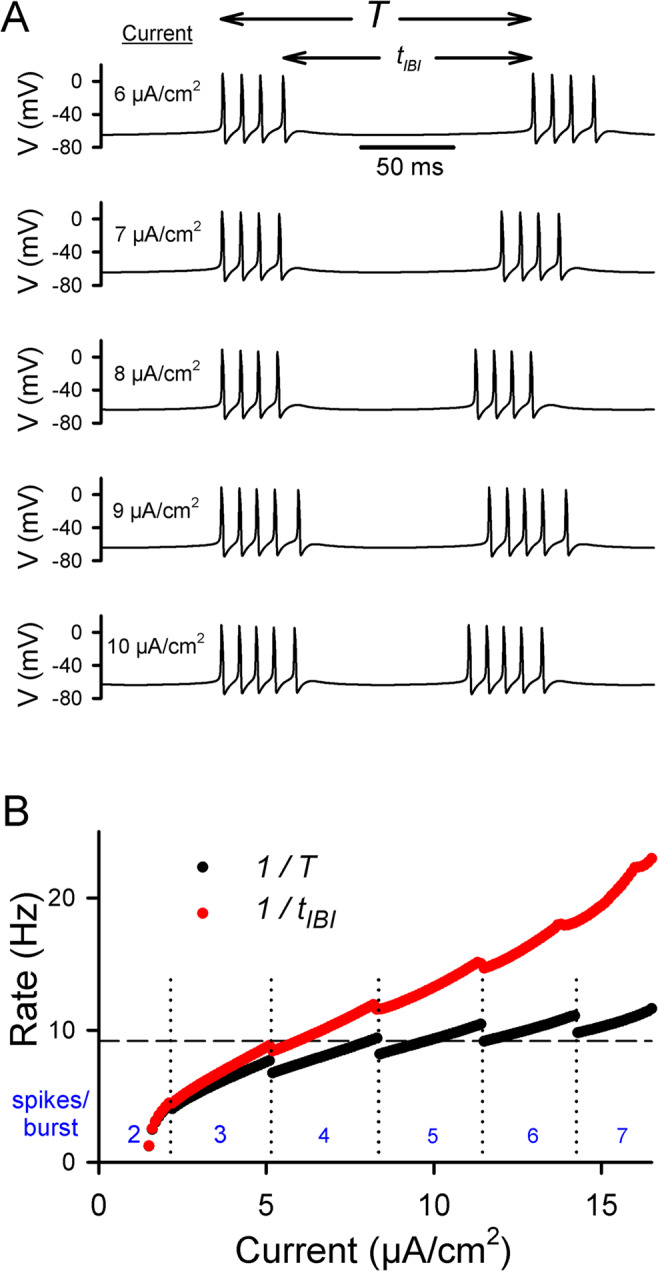
Response of the circle/circle bursting model neuron to a constant current stimulus. (**A**) Voltage traces for constant current stimuli ranging from 6 to 10 µA/cm^2^. The burst period *T* associated with a burst was defined as the time between its first spike and the first spike of the preceding burst. The interburst interval *t*_IBI_ associated with a burst was defined as the time between its first spike and the last spike of the preceding burst. (**B**) Plots of the inverses of the burst period and the interburst interval as a function of the (constant) stimulus for currents ranging from 1.5 to 16.5 µA/cm^2^. The vertical dotted lines specify the values of the current at which the number of spikes in the burst changed. The horizontal dashed line corresponds to a period (109 ms) that could be generated by three different values of the current density

**Fig. 2:**
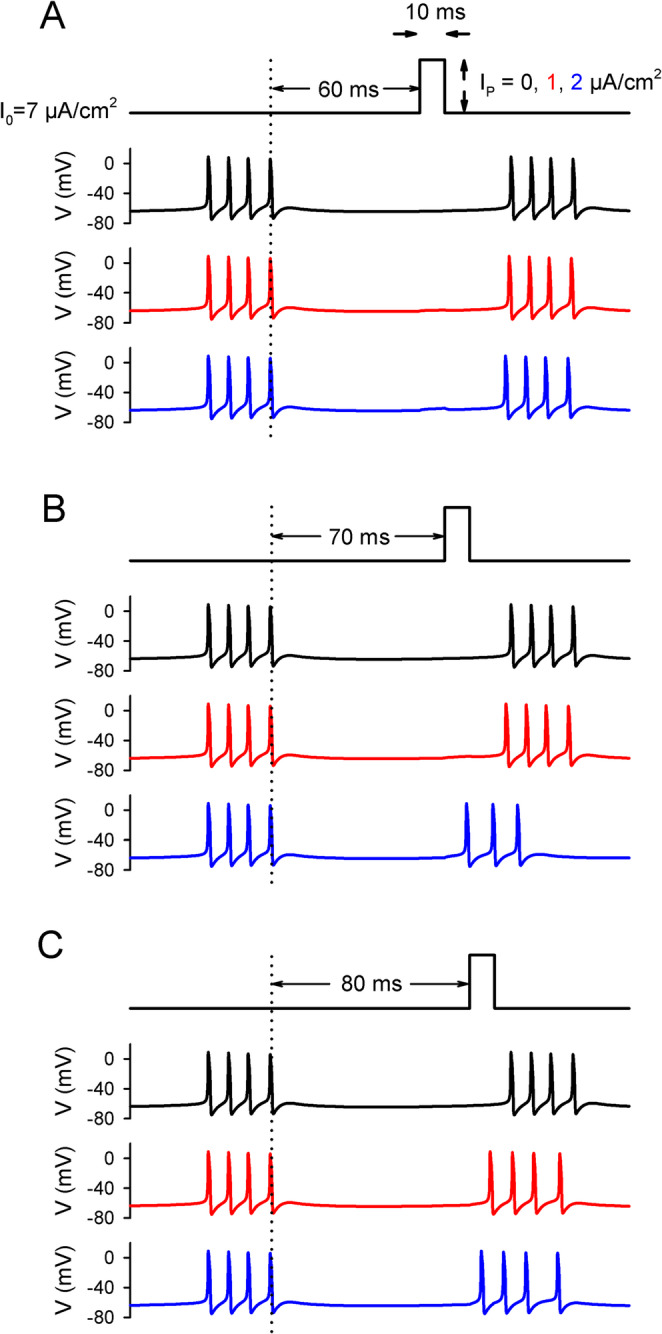
Response of the bursting model neuron to a short excitatory input. (**A**) Voltage traces in response to a 10 ms-duration current pulse with amplitude 0 (black), 1 (red), and 2 µA/cm^2^ (blue) injected 60 ms after the end of the previous burst. The baseline current was 7 µA/cm^2^. (**B**) The response to pulses injected 70 ms after the end of the previous burst. (**C**) The response to pulses injected 80 ms after the end of the previous burst. Note that the black voltage traces in A-C are identical

**Fig. 3:**
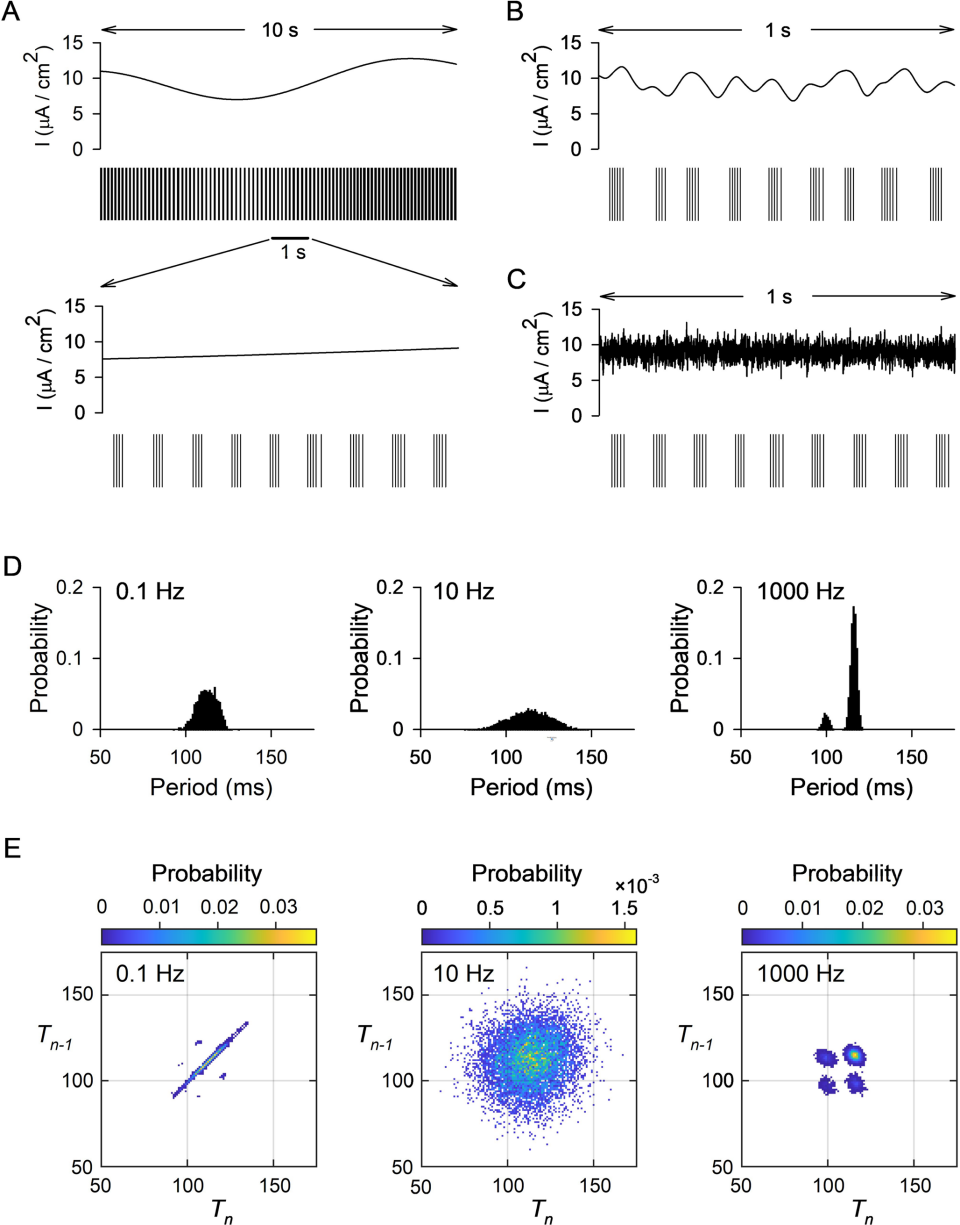
Response of the bursting model neuron to stimuli constructed from white noise low-pass filtered at three different frequencies (0.1 Hz, 10 Hz, 1000 Hz). In each case, the duration of the stimulus was 1000 s, the dc amplitude of the stimulus was 9 µA/cm^2^ and the rms amplitude of the time-varying portion of the stimulus was 1 µA/cm^2^. (**A**) *Top traces*. A 10-s portion of the neuron’s response (extracted spike times) to the stimulus that had been low-pass filtered at 0.1 Hz. *Bottom traces*. An expanded view of a 1-s portion of the stimulus and response. (**B**) A 1-s portion of the response to the stimulus filtered at 10 Hz. (**C**) A 1-s portion of the response to a stimulus filtered at 1000 Hz. (**D**) Probability histogram of the burst periods (1 ms bins) produced during 1000-s stimuli filtered at the three different frequencies. *Left*. Filter frequency 0.1 Hz, 8949 burst periods (corresponding to 8950 bursts). *Center*. Filter frequency 10 Hz, 8827 burst periods. *Right*. Filter frequency 1000 Hz, 8808 burst periods. (**E**) *Left*. Heat map showing the probability of pairs of consecutive burst periods (1 ms × 1 ms bins) generated during the 0.1 Hz stimulus (8948 pairs). The vertical axis corresponds to the first of the two periods; the horizontal to the second. *Center*. Probability of consecutive burst periods for the 10 Hz stimulus. *Right*. Probability of consecutive burst periods for the 1000 Hz stimulus

**Fig. 4:**
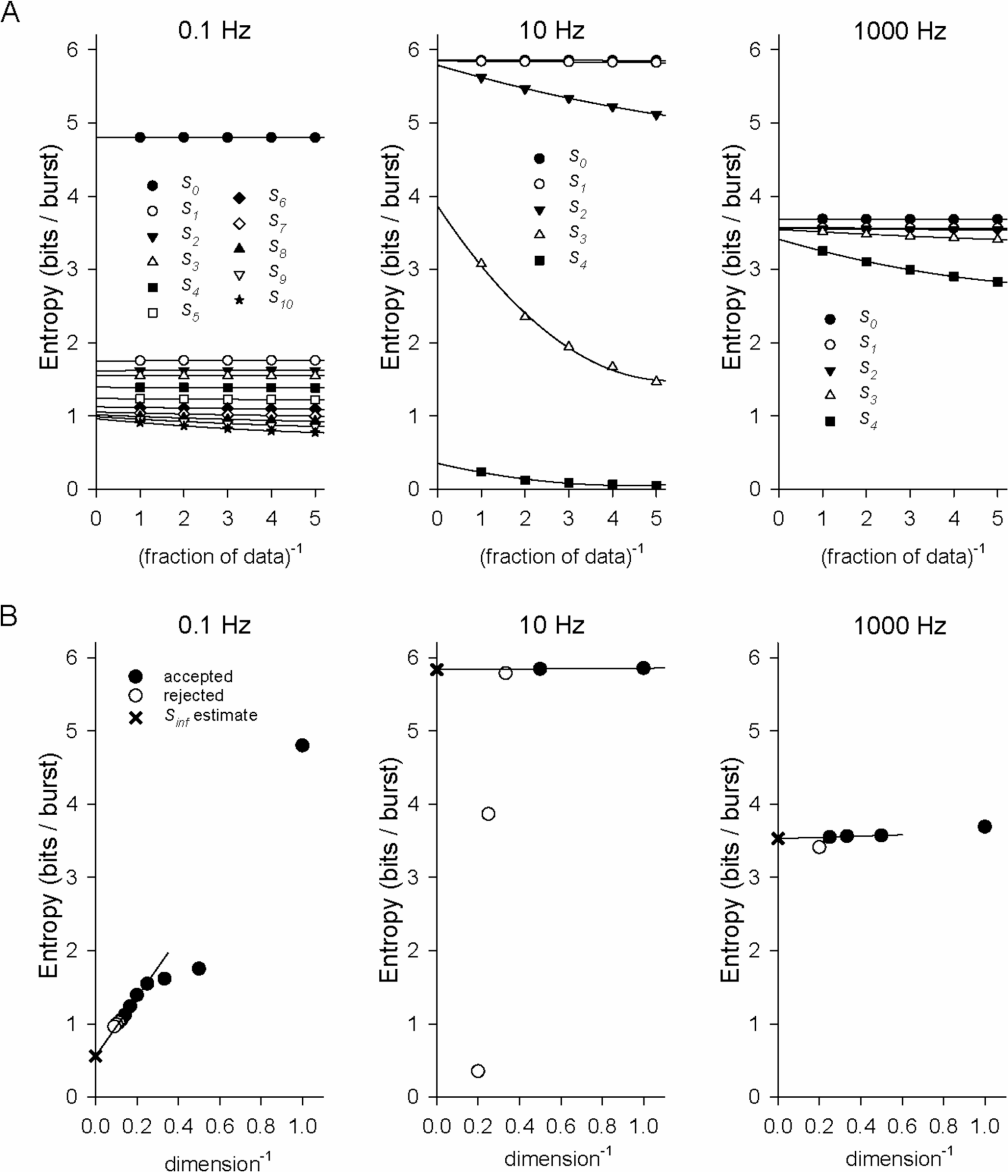
Estimation of entropy associated with the timing of bursts (periods) produced in response to stimuli constructed from white noise low-pass filtered at three different frequencies (0.1 Hz, 10 Hz, 1000 Hz). This was done by calculating the upper bounds on the entropy *S*_*i*_ using portions of the data set. (**A**) *Left*. Plot of *S*_0_, *S*_1_,…*S*_10_ calculated from burst periods in response to a stimulus filtered at 0.1 Hz (dc amplitude 9 µA/cm^2^ and rms amplitude 1 µA/cm^2^ as in Fig. 3, total duration 432,000 s) using the entire data set, 1/2 of the data, 1/3 of the data, 1/4 of the data, and 1/5 of the data (symbols) versus the inverse of that fraction. The curves through the data points are quadratic fits, and the intercepts with the vertical axis are the estimate of the *S*_*i*_ for an infinitely large data set. *Center*. *S*_0_…*S*_4_ calculated from burst periods in response to a stimulus filtered at 10 Hz (with amplitudes as in Fig. 3, duration 180,000 s) using fractions of the data set plotted versus inverse fraction, along with quadratic fits. *Right*. *S*_0_…*S*_4_ calculated from burst periods in response to a stimulus filtered at 1000 Hz (with amplitudes as in Fig. 3, duration 300,000 s) using fractions of the data set plotted versus inverse fraction, along with quadratic fits. (**B**) *Left*. Plot of the estimated *S*_0_…*S*_10_ for an infinite train of bursts produced in response to a stimulus filtered at 0.1 Hz (intercepts in plot immediately above) versus the inverse of the dimension, where the dimension associated with *S*_*i*_ is *i* + 1. The solid circles indicate that the difference between the estimate of *S*_*i*_ for an infinite data set and the value using the entire finite data set was less than 2% of the estimated *S*_*i*_, in this case *S*_0_…*S*_7_. The open circles are the estimated *S*_*i*_ that failed the test (*S*_8_…*S*_10_) and were discarded from the analysis. The line is a linear fit through *S*_3_…*S*_7_. The intercept with the vertical axis, marked with an ×, is *S*_*inf*_, corresponding to infinite dimension, and our best estimate of the entropy. *Center*. Estimated *S*_0_…*S*_4_ for an infinite train of bursts produced by a stimulus filtered at 10 Hz (intercepts in plot above). *S*_*inf*_ calculated by fitting a line through *S*_0_ and *S*_1_. *Right*. Estimated *S*_0_…*S*_4_ for an infinite train of bursts produced by a stimulus filtered at 1000 Hz (intercepts in plot above). *S*_*inf*_ calculated by fitting a line through *S*_1_…*S*_3_

**Fig. 5:**
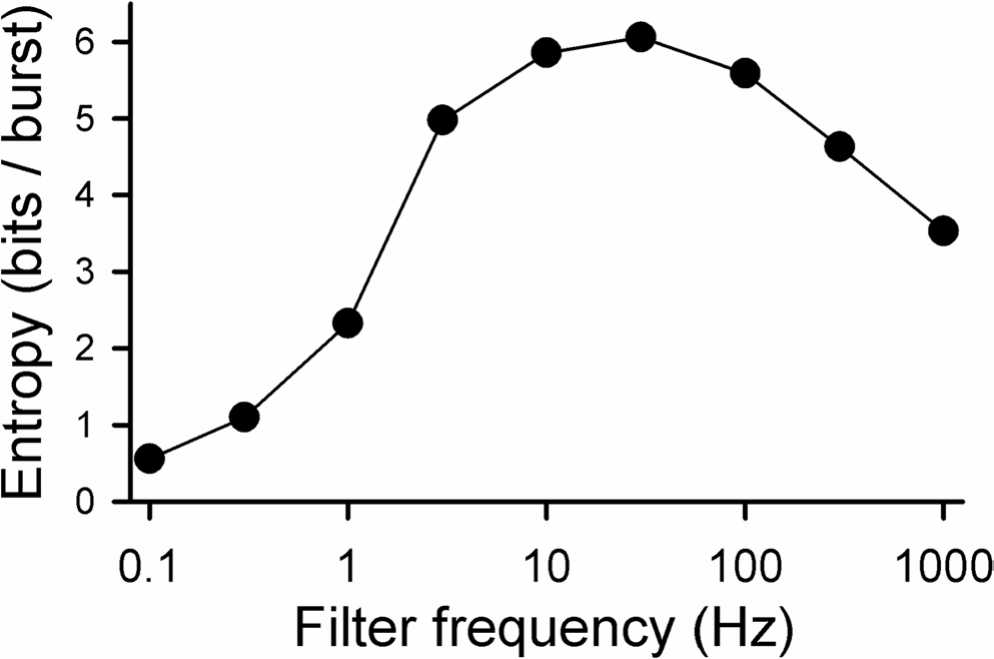
Entropy per burst as a function of filter frequency. Each data point is the value of *S*_*inf*_, calculated as in Fig. 4, for filter frequencies of 0.1, 0.3, 1, 3, 10, 30, 100, 300 and 1000 Hz. In each case, the stimulus had a dc amplitude of 9 µA/cm^2^, and the time-varying component had a rms amplitude of 1 µA/cm^2^. Stimulus durations ranged from 120,000 s (3 Hz) to 432,000 s (0.1 Hz)

**Fig. 6:**
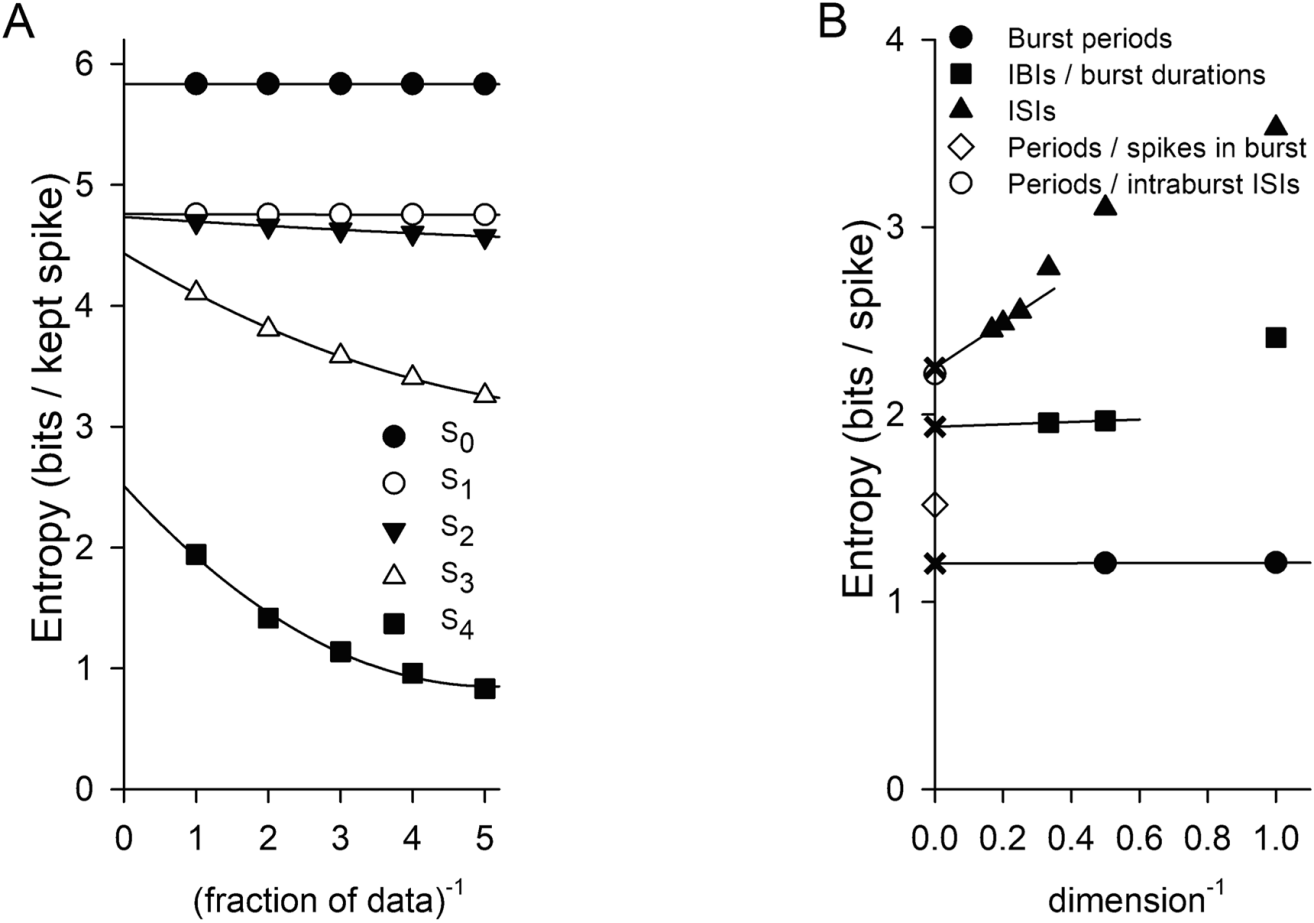
Entropy per spike for the bursting model neuron calculated using intervals and by using Eq. 9. (**A**) Plot of *S*_0_…*S*_4_ (bits / kept spike) calculated from burst durations and IBIs in response to a stimulus filtered at 10 Hz (dc amplitude 9 µA/cm^2^, rms amplitude 1 µA/cm^2^, stimulus duration 180,000 s) versus the inverse fraction of the data used. The curves are quadratic fits, and the intercepts are the estimates of *S*_*i*_ for an infinitely large data set. (**B**) Entropy per spike associated with a burst train (same stimulus characteristics as in A) versus inverse dimension when periods, IBIs and burst durations, or all ISIs were considered, and from Eq. 9 by considering the number of spikes in a burst or the sequences of intraburst ISIs. For the ISI experiments, the stimulus was 480,000 s long. The burst period data are from Fig. 4B (center) and were converted from bits/burst to bits/spike by dividing by 4.848, the average number of spikes per burst. The IBI / burst duration data from Fig. 6A were converted to bits/spike by dividing by half this number, 2.424. To obtain *S*_*inf*_, indicated by the × on the vertical axis, linear fits were made to *S*_0_ and *S*_1_ (burst periods), to *S*_1_ and *S*_2_ (IBIs / burst durations), and to *S*_3_…*S*_5_ (ISIs). The open diamond and the open circle are the entropies calculated using Eq. 9 in the cases when the number of spikes in a burst or the intraburst ISI sequences, respectively, were considered for a stimulus 100,000 s long

**Fig. 7:**
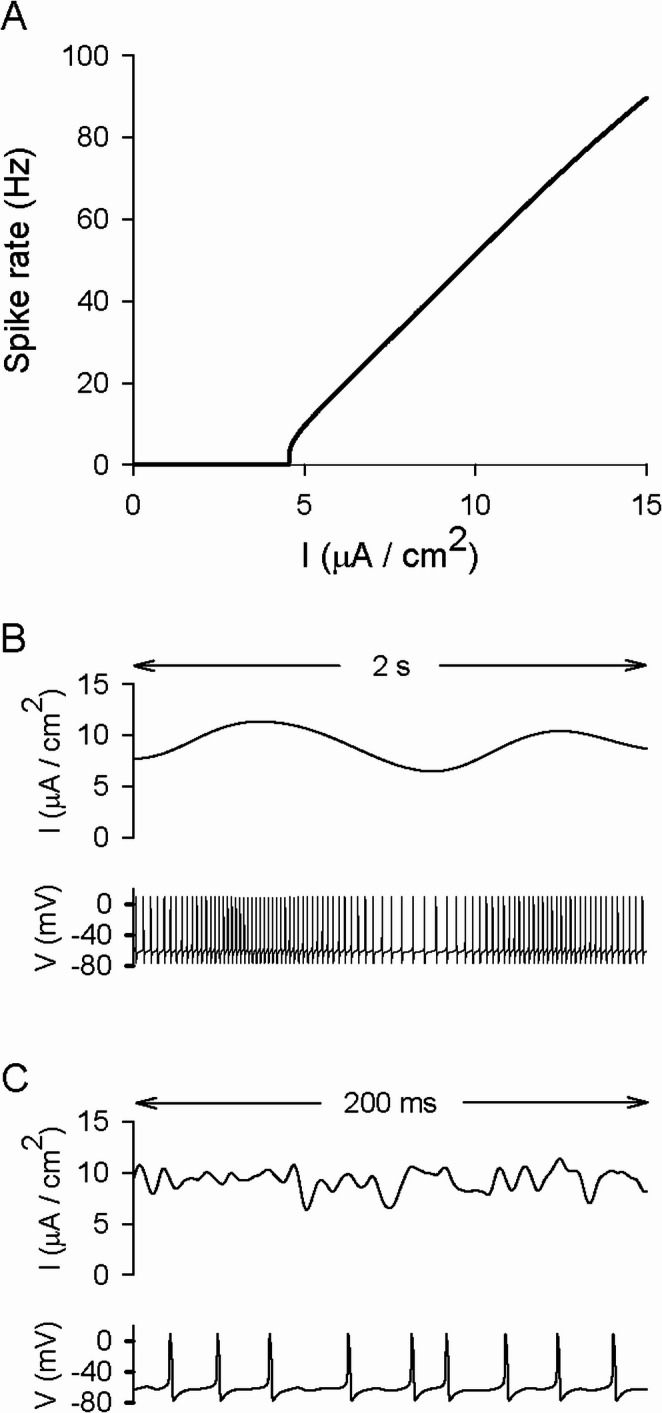
Response of the model neuron when the conductances were modified so that it no longer produced bursts. The circle/circle bursting neuron became a spiking neuron with the same average spike rate (~ 43 Hz) when the slow sodium maximal conductance was set to zero and the slow potassium maximal conductance was reduced to 4.5 mS/cm^2^. (**A**) Spike rate plotted as a function of a constant current stimulus. (**B**) A portion of a stimulus (dc amplitude 9 µA/cm^2^, rms amplitude 1 µA/cm^2^) filtered at 1 Hz (top) and a voltage trace showing the spiking neuron’s response (bottom). (**C**) A portion of a stimulus (dc amplitude 9 µA/cm^2^, rms amplitude 1 µA/cm^2^) filtered at 100 Hz (top) and the spiking neuron’s response (bottom)

The results shown in Figs. 1, 2, 3 and 7, and 9 were computed using MATLAB (releases R2022b – R2023b) (MathWorks, Natick, MA). Code for the entropy and information calculations shown in Figs. 4, 5 and 6 (except 6b), 8, and 10 was written in Python (release 3.9.18) and run on the Wiegand Advanced Visualization Environment (WAVE) HPC Center at Santa Clara University. (Fig. 6b has both MATLAB and WAVE results.) The default maximum job time allowed on WAVE was set at four days, but jobs were kindly extended to up to a week for some calculations.

## Results

### Rate and timing of bursting

The bursting model neuron described in the Methods section was quiescent in the absence of a stimulus but began to burst when injected with a constant current density of just under 1.5 µA/cm^2^. As the magnitude of the constant current density increased, the neuron produced more frequent bursts that contained more spikes (Fig. 1A). The relationship between the period *T* (or its inverse, the average burst rate) and the stimulus amplitude (black dots) was not one to one over the entire range of inputs (Fig. 1B). For example, there were three different current densities that resulted in bursting with a period of 109 ms (burst rate = 9.17 Hz) (Fig. 1B, dashed horizontal line); knowing the number of spikes in the burst (4, 5 or 6) was also required to determine the stimulus. In contrast, the interburst interval *t*_IBI_ (or its inverse) could more often be related to a unique stimulus amplitude (Fig. 1B).

Figure 2 shows how the response of the model neuron to a short (10 ms), excitatory input depended both on the timing and the amplitude of the input. When the pulse was delivered too soon (60 ms) after the preceding burst (Fig. 2A), the result was merely an amplitude-dependent change (1–2 ms) in the start time of the next burst. As the delay between the end of the previous burst and the pulse was increased, the minimum stimulus amplitude required to elicit a burst itself decreased (Fig. 2B, C).

Figure 3 shows and analyzes the response of the neuron to current stimuli constructed from white noise that was low-pass filtered (4th-order Butterworth) at one of three different frequencies: 0.1 Hz, 10 Hz and 1000 Hz. In each case the mean of *I*(*t*) (i.e., its dc component) was 9 µA/cm^2^, the rms amplitude of the time-varying component was 1 µA/cm^2^, and the duration was 1000 s. Portions of each stimulus and extracted burst trains are plotted in Fig. 3A-C. Probability histograms of the burst periods elicited during each of the stimuli are shown in Fig. 3D. The number of bursts (and hence periods) elicited in each case were nearly identical, but their distributions were very different – broadest for the 10 Hz stimulus and narrowest for 1000 Hz. In the latter case, there were two discrete peaks in the histogram; 86% of the periods were found in the taller distribution that had a maximum at 116 ms, identical to the period (rounded to the nearest ms) when only a 9 µA/cm^2^ dc signal was injected into the neuron (Fig. 1B). The smaller peak in the histogram can be explained by considering the burst train shown in Fig. 3C. Most of the bursts had five spikes; occasionally one had four spikes (here the fourth one), and the subsequent burst period was shorter. Figure 3E shows probability heat plots of pairs of consecutive burst periods produced in response to the three stimuli. For the 0.1 Hz stimulus, the period varied little, burst to burst, and points clustered mainly near the line *Τ*_*n*−1_ = *Τ*_*n*_, except when the number of spikes in the burst occasionally changed and the following period was affected (e.g., the period preceding the seventh burst in Fig. 3A bottom). For the two higher frequency stimuli, the probability *P*(*T*_*n*_,*Τ*_*n*−1_) appeared to be approximately equal to the product of *P*(*Τ*_*n*_) and *P*(*T*_*n*−1_) (Fig. 3D), indicating that the values of consecutive periods were far less correlated than for the 0.1 Hz stimulus.

### Entropy of bursts (periods) produced by low-pass filtered white noise

The results shown in Fig. 3 suggested that the entropy associated with the period of burst trains produced by a low-pass filtered white-noise stimulus is maximized at some frequency between 0.1 Hz and 1000 Hz. For the 0.1 Hz stimulus, the periods changed very slowly and were correlated over many bursts, reducing the entropy. For the 1000 Hz stimulus, consecutive periods were far less correlated, but the distribution of periods was much narrower. The periods in response to the 10 Hz stimulus varied widely in their length and consecutive pairs appeared largely uncorrelated, and so we expected that the entropy associated with this response should be the largest of the three.

Calculation of an upper bound on the entropy, *S*_*i*_, requires a sufficiently large data set such that the associated probabilities used in Eqs. 6–8 and their extensions are adequately represented. Accurate estimation of the true entropy *S*_*inf*_ requires that enough *S*_*i*_ be obtained so that an extrapolation that accounts for correlations can be trusted. Figure 4A shows calculations of *S*_*i*_ using long data sets of burst trains produced by white-noise stimuli that were low-pass filtered at 0.1 Hz (*left*), 10 Hz (*center*), or 1000 Hz (*right*). The data points are calculations using fractions of the data set, and the curves are quadratic fits. The intercepts with the vertical axis, corresponding to an infinitely long data set, are the estimates of *S*_*i*_; the flatter the curve, the more trustworthy the extrapolation was. For the 10 Hz data set, *S*_0_ and *S*_1_ differed by less than 0.16%, indicating that consecutive periods were uncorrelated. For the 1000 Hz set, the value of *S*_1_ and *S*_3_ differed by less than 0.7%, indicating no correlation beyond consecutive periods. For the 0.1 Hz set, however, the correlations extended over many bursts.

Figure 4B illustrates how the calculated *S*_*i*_ were used to obtain *S*_*inf*_, which accounts for infinite correlations and hence is the best estimate of the true entropy. For the 0.1 Hz (*left*), 10 Hz (*center*), and 1000 Hz (*right*) data sets shown above in Fig. 4A, the calculated values of *S*_*i*_ are plotted vs. 1/(*i* + 1), the inverse of the dimension. Only the values of *S*_*i*_ that met the “2% criterion” (Methods) (solid circles) were deemed sufficiently accurate to keep; those that did not (open circles), although plotted, were not used in the analysis. A linear fit to some or all of the solid circles was used to compute *S*_*inf*_ (shown as a ×), the intercept with the vertical axis and corresponding to infinite dimension. Values of *S*_*i*_ for smaller values of *i* that clearly did not fall on a line through the higher-dimensional points were excluded (Fig. 4B left). While nearly horizontal lines through *S*_0_ and *S*_1_ (for 10 Hz) and through *S*_1_*…S*_3_ (for 1000 Hz) were used to find *S*_*inf*_, simply taking *S*_*inf*_ = *S*_*0*_ and *S*_*inf*_ = *S*_1_, respectively, would have been sufficient for our purposes. For the 0.1 Hz data set, *S*_0_…*S*_7_ met the 2% criterion. For the extrapolation, we chose to fit *S*_3_…*S*_7_ to the line and obtained a value of 0.56 bits/burst for *S*_*inf*_. (Fitting a line only to *S*_5_…*S*_7_ resulted in a value of 0.50 bits/burst for *S*_*inf*_. Using *S*_2_…*S*_7_, which clearly do not all fall on a line, yielded 0.76 bits/burst.) Obtaining acceptable estimates of *S*_*i*_ for larger values of *i* was easiest for 0.1 Hz, because the correlations resulted in very narrow joint probability distributions (e.g., Fig. 3E *left*), requiring less data to approximate accurately; conversely, the task was most difficult for 10 Hz (only *S*_0_ and *S*_1_ calculated acceptably), for which the probability distributions were broadest (Fig. 3D *center*, Fig. 3E *center*). As expected, *S*_*inf*_ associated with burst periods produced by the signal filtered at 10 Hz (5.84 bits/burst) was larger than that for the 0.1 or 1000 Hz data sets (0.56 and 3.53 bits/burst, respectively).

Figure 5 plots the entropy *S*_*inf*_ in bits/burst associated with burst trains produced in response to a stimulus filtered at nine frequencies ranging from 0.1 to 1000 Hz. The values for 0.1, 10, and 1000 Hz were taken from Fig. 4; the entropies for the other six frequencies were obtained in a similar manner (data not shown). For 0.1, 0.3, and 1 Hz, *S*_*i*_ appeared still to be decreasing for the highest dimension value of *S*_*i*_ that was accurately obtained (*S*_7_, *S*_4_ and *S*_3_, respectively). For 3, 10, and 30 Hz, *S*_0_ was a good estimate of the entropy; for 100, 300, and 1000 Hz, *S*_1_ was a good estimate. The largest entropy (6.06 bits/burst), corresponded to the response to a stimulus filtered at 30 Hz, which was a factor of ~ 3.4 larger than the average burst frequency. For comparison, the Poisson result, ${log}_{2}\left(\frac{e}{r\varDelta{t}}\right)$ (MacKay & McCulloch, 1952) is ~ 8.2 bits/burst for a comparable rate (~ 9 Hz) and Δ*t* = 1 ms.

### Entropy when additional spikes considered

Ultimately, we were interested in how much each spike in the burst train contributes to the entropy. To calculate the entropy/spike for the burst trains, the entropy/burst data plotted in Fig. 5 must be divided by the average number of spikes per burst, which varied from 4.58 (30 Hz) to 4.88 (1000 Hz). The coarsest correction to treating the bursts as unitary objects is to consider their duration. Instead of periods, we thus considered a series of intervals that alternated between longer IBIs and shorter burst durations when calculating the entropy. We expected that including the last spike of each burst in our analysis, in addition to the first one, would result in a larger entropy/spike associated with the burst train, because any particular period could correspond to multiple combinations of an IBI and a duration. We tested this hypothesis using a signal filtered at 10 Hz. Figure 6A shows calculations of *S*_*i*_ per “kept” spike (two per burst) using IBIs and durations in a long data set. Again, the data points are calculations using fractions of the data set, the curves are quadratic fits, and the intercepts with the vertical axis are the estimates of *S*_*i*_. Figure 6B plots entropy per spike versus inverse dimension (solid symbols) for burst trains produced by a 10 Hz signal for three cases: (1) considering only the first spike in each burst (periods), (2) considering the first and last spike (IBIs and durations), and (3) considering every spike (ISIs). In all cases, only *S*_*i*_ that met the 2% criterion are shown. The period data are replotted from Fig. 4B (center) and were converted from bits/burst to bits/spike by dividing by 4.848, the average number of spikes per burst. The IBI / duration data are the intercepts from Fig. 6A divided by half this number, 2.424, the average number of total spikes per “kept” spike. For the IBI / duration data, a linear fit through *S*_1_ and *S*_2_ was nearly horizontal, indicating that *S*_1_ was a good estimate of the entropy. Given that consecutive periods were uncorrelated, it is not surprising that consecutive IBIs or durations (which are considered in the calculation of *S*_2_) appeared to be also. The best estimate of *S*_*inf*_, which is marked in each case by an × on the axis, was approximately 1.6 times larger for IBIs / durations than for periods (1.94 bits/spike versus 1.21 bits/spike). Accounting for all spikes and the corresponding ISIs must yield an entropy per spike larger than when considering IBIs / durations, because any given burst duration could be comprised of multiple combinations of ISIs. However, most ISIs within a burst were 7, 8, or 9 ms, and so we expected the additional contribution to entropy to be modest. In Fig. 6B, *S*_0_… *S*_5_ are plotted for ISIs, as is a linear fit to *S*_3_… *S*_5_, which yielded an estimate of *S*_*inf*_ of 2.25 bits/spike. From these data alone, it was not clear to us if the *S*_*i*_ had flattened out or would continue to decrease for *i ≥* 6, but, in any case, *S*_*inf*_ could be no greater than *S*_5,_ 2.45 bits/spike.

Because, for this stimulus, the start times of bursts were independent of the preceding ones, we were also able to estimate the entropy when accounting for all spikes, by considering the joint probability $P\left({\tau}_{n},{b}_{n}\right)$ of periods and the spiking within bursts. When *b*_*n*_ described the number of spikes in a burst (or equivalently the number of ISIs), Eq. 9 yielded 1.52 bits/spike, an increase of only 0.31 bits/spike above the result when only periods were considered (open diamond in Fig. 6B). This was not surprising, as 97.3% of bursts had four, five, or six spikes. The Spearman’s rank correlation coefficient ρ between the period and the number of spikes in a burst was measured to be 0.247, weakly positive. Naively assuming independence (i.e. zero correlation) between period and spike number resulted only in a small error in the calculated entropy (0.67% larger using Eq. 10). In response to this stimulus, 636 distinct intraburst ISI sequences (precision Δ*t* = 1 ms) were observed. The three most probable sequences (in ms) were {8, 8, 9} (11.1%), {8, 7, 8, 9} (10.2%), and {8, 7, 7, 8} (9.4%). The ten most probable ISI sequences accounted for 64.7% of all sequences, and the twenty most probable accounted for 82.9%. When *b*_*n*_ described these sequences, Eq. 9 yielded an entropy of 2.22 bits/spike (open circle), within 1.5% of the value of *S*_*inf*_ obtained from the linear extrapolation when considering all ISIs (Fig. 6B), providing confidence in both calculations. Using Eq. 10, instead, again resulted only in a small error in entropy (0.80% larger).

### Information transmission by bursting and spiking neurons

Bursting by the circle/circle neuron results from the activation and deactivation of the slow sodium and slow potassium conductances. When the slow sodium conductance is removed, the ability to burst disappears. Figure 7A is a plot of spike rate versus the amplitude of a dc stimulus when ${\stackrel{-}{g}}_{\mathrm{N}\mathrm{a},\mathrm{s}\mathrm{l}\mathrm{o}\mathrm{w}}$ was changed from 2 to 0 mS/cm^2^ and ${\stackrel{-}{g}}_{\mathrm{K}\left(\mathrm{M}\right)}$ was reduced from 20 to 4.5 mS/cm^2^. For a 9 µA/cm^2^ stimulus, the spike rates of this spiking neuron and the bursting neuron were almost exactly the same (~ 43 Hz). Figure 7B shows the response of the spiking neuron to a portion of a stimulus that consisted of 9 µA/cm^2^ dc and 1 µA/cm^2^ (rms) low-pass filtered (1 Hz) white noise. The timing of spikes exhibited long-range correlations, with the spike rate approximately tracking the stimulus. Figure 7C shows the response when the amplitudes were the same, but the time-varying portion of the stimulus was instead filtered at 100 Hz. In this case, individual spikes appeared to be elicited by features in the stimulus.

**Fig. 8:**
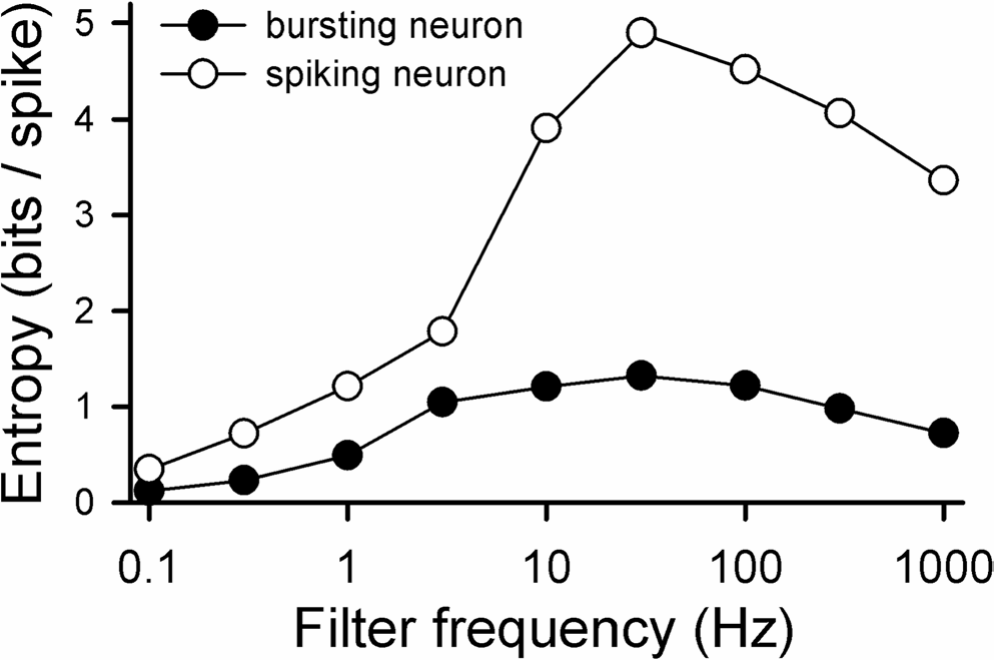
Entropy per spike as a function of filter frequency for the bursting model neuron using periods and the spiking model neuron using ISIs. In each case, the stimulus had a dc amplitude of 9 µA/cm^2^, and the time-varying component had a rms amplitude of 1 µA/cm^2^. The entropy per spike for the bursting neuron was obtained by dividing the numbers plotted in Fig. 5 by the average number of spikes per burst for each filter frequency. For the spiking neuron calculations, stimulus durations ranged from 36,000 to 120,000 s

Upper bounds on the entropy associated with a spike train produced by the spiking neuron can similarly be calculated from ISIs using Eqs. 6–8 and their extensions. As was done with the bursting neuron, the entropy *S*_*inf*_ for an infinite data set when considering infinite correlations was estimated from the calculated *S*_*i*_. Figure 8 shows the dependence of entropy/spike on the filter frequency for both the spiking neuron (considering ISIs) and the bursting neuron (considering periods). As in Fig. 5, the stimulus had a dc amplitude of 9 µA/cm^2^, and the time-varying component had a rms amplitude of 1 µA/cm^2^. For the bursting neuron, as in Fig. 6B, the number of spikes per burst was used to convert bits/burst to bits/spike. There are two comments to make. First, the entropy/spike for the spiking neuron was larger than for the bursting neuron for all stimuli. This is not surprising—nearly 80% of the spikes in the burst trains were ignored in the calculations. Second, and more interesting, the entropy/spike increased dramatically for the bursting neuron between 1 and 3 Hz; a similar increase occurred at a higher frequency for the spiking neuron—between 3 and 10 Hz. In response to the 3 Hz stimulus, we had observed that consecutive burst periods were uncorrelated; however, consecutive ISIs produced by the spiking neuron were not (*S*_2_/*S*_0_ = 0.57).

Based on Fig. 8, it seemed clear that the entropy/spike associated with the response of the spiking neuron would always be larger than that of the bursting neuron for any time-varying stimulus consisting of combinations of waveforms low-passed filtered at frequencies between 0.1 and 1000 Hz. We wondered, however, if there might be a situation in which the bursting neuron could provide more information/spike about a noisy stimulus than the spiking neuron could. This might occur if the bursting neuron did a better job than the spiking neuron of capturing features of the good part of the stimulus (“signal”) and was also less responsive to the “noise” corrupting the signal. Looking at Fig. 8, we speculated this might occur if the signal were white noise filtered at 3 Hz and the noise were white noise filtered at 1000 Hz. For the bursting neuron, *S*_3Hz_ / *S*_1000Hz_ = 1.41, while for the spiking neuron the ratio was 0.53.

**Fig. 9:**
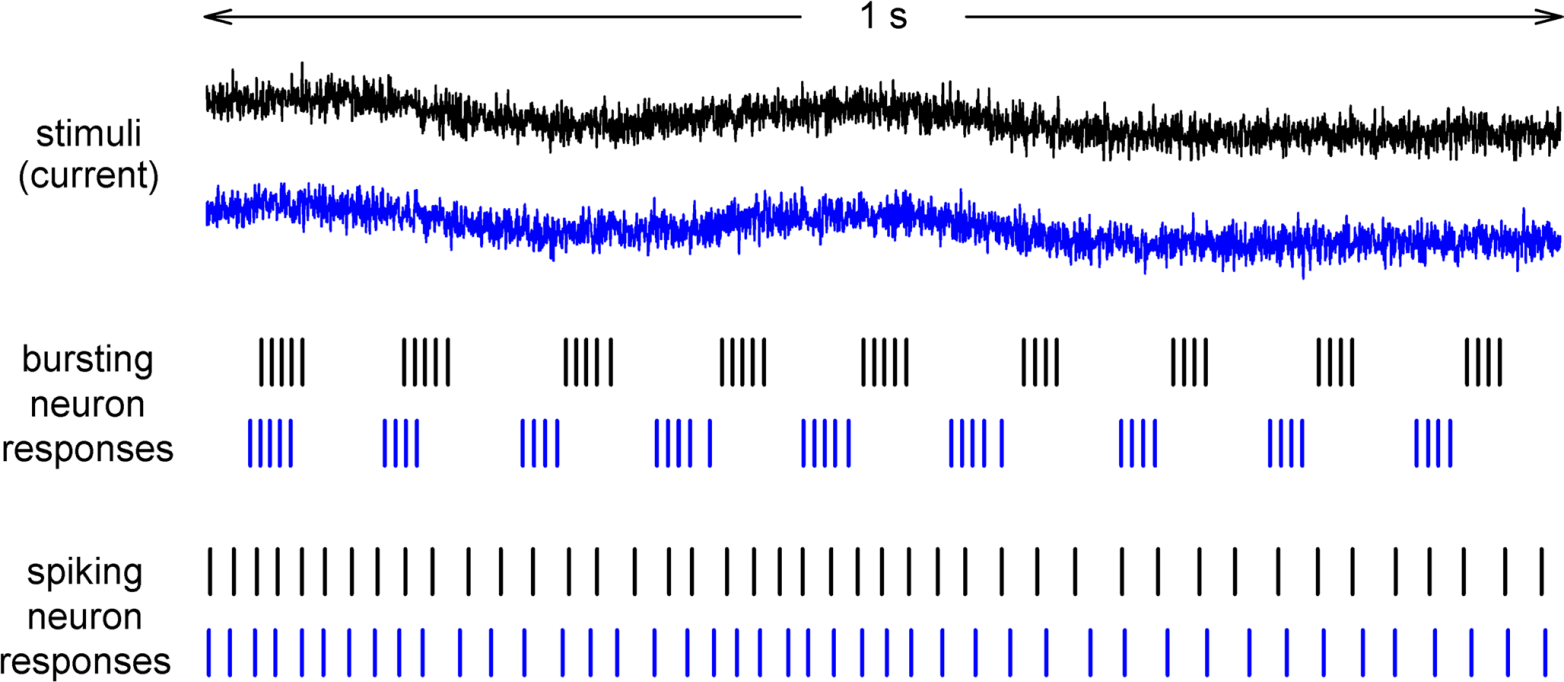
Response of the bursting and spiking neuron to a stimulus that consisted of a repeated signal to which noise had been added. *Top*. Each of the two stimuli (black and blue) was composed of a dc component (9 µA/cm^2^), the same time-varying component low-pass filtered at 3 Hz (“the signal”) (rms 0.707 µA/cm^2^), and different time-varying components filtered at 1000 Hz (“the noise”) (rms 0.707 µA/cm^2^). *Center.* The corresponding responses of the bursting neuron to the two stimuli. *Bottom*. The corresponding responses of the spiking neuron to the two stimuli

Figure 9 shows 1-s portions of responses of the bursting neuron and spiking neuron to stimuli that consisted of a dc component (9 µA/cm^2^), a 3 Hz signal (rms 0.707 µA/cm^2^), and two different realizations of 1000 Hz noise (rms 0.707 µA/cm^2^). (The rms of the time-varying portion of a stimulus was thus again 1 µA/cm^2^.) The 3 Hz signal component of the stimuli was the same for the black and blue traces (*top*), but the 1000 Hz component was different. The two corresponding responses of the bursting neuron (*center*) and spiking neuron (*bottom*) are shown below. In each case, the neuron was reporting something about the stimulus, but whether information was being transmitted efficiently could not be determined by eye; a calculation was required.

**Fig. 10:**
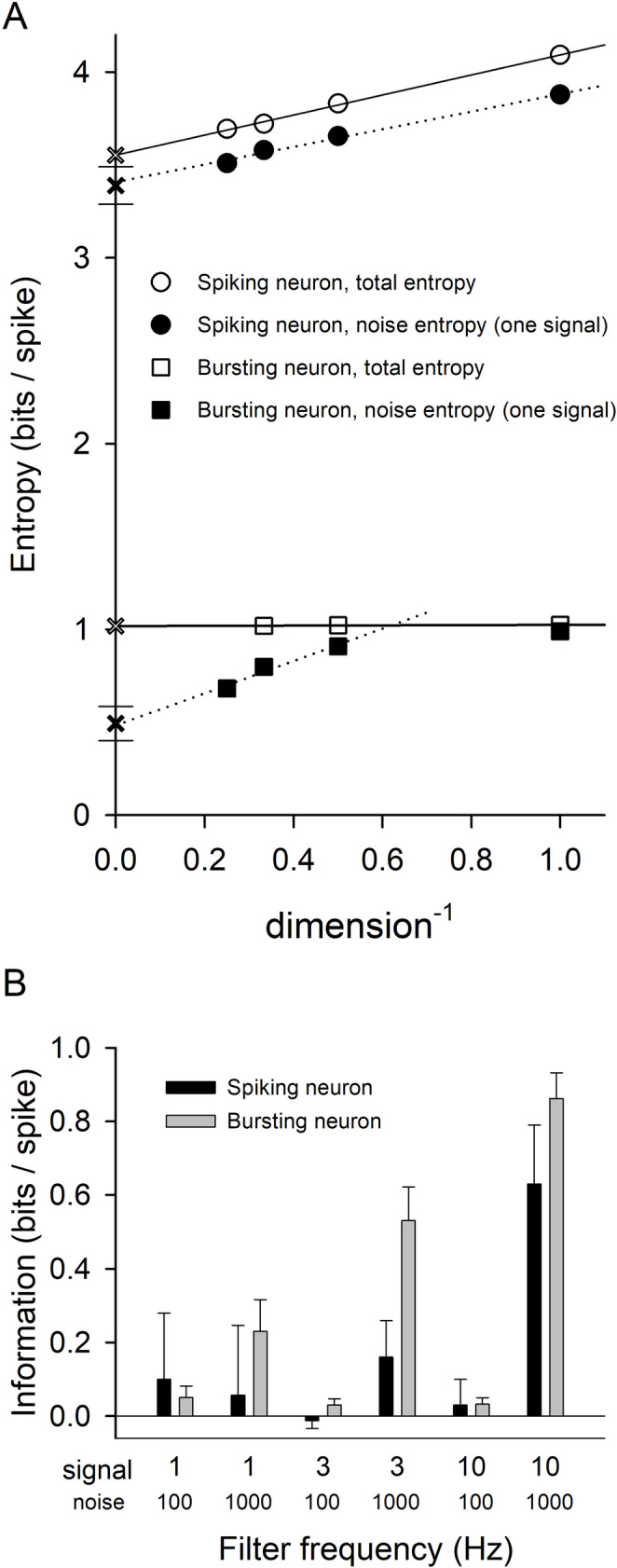
Calculation of total entropy, noise entropy and information associated with responses of the spiking and bursting neurons to a stimulus that consisted of a repeated signal to which noise had been added. For the bursting neuron, this was done using periods; for the spiking neuron, ISIs were used. (**A**) The stimulus used was the same as in Fig. 9: a dc component (9 µA/cm^2^), a time-varying component filtered at 3 Hz (rms 0.707 µA/cm^2^), and a time-varying component filtered at 1000 Hz (rms 0.707 µA/cm^2^). Total entropy was computed from long simulations (180,000 s) in which the time-varying components were randomly constructed. The *S*_*i*_ that met the 2% criterion are plotted versus inverse dimension in open symbols. Linear fits to *S*_0_…*S*_3_ and *S*_0_…*S*_2_ were used to find *S*_*inf*_ for the spiking and bursting neuron, 3.55 and 1.02 bits/spike respectively. (Fitting to *S*_1_…*S*_3_ and *S*_1_ */ S*_2_ left these numbers unchanged.) On average there were 4.768 spike per burst. The value of *S*_*inf*_ in each case is indicated by an open × on the vertical axis. Noise entropy was computed from repeated 1-s trials in which the 3 Hz signal was the same each time but the 1000 Hz noise was random. This was done for ten different 3 Hz signals (360,000 or 420,000 trials). The *S*_*i*_ that met the 2% criterion are plotted versus inverse dimension. Linear fits to *S*_0_…*S*_3_ and *S*_1_…*S*_3_ were used to find *S*_*inf*_ for the spiking and bursting neuron, respectively. These are shown on the graph for one of the ten 3-Hz signals for each neuron. The average values of *S*_*inf*_ for the spiking and bursting neurons are each indicated by a solid × on the vertical axis, 3.39 and 0.49 bits/spike respectively. (Fits to *S*_1_…*S*_3_ and *S*_2_ */ S*_3_ instead yielded average values of 3.37 and 0.44 bits/spike, respectively.) The error bars above and below the average *S*_*inf*_ indicate ± one standard deviation. Data for the two signals that are shown were chosen because their values of *S*_*inf*_ were each close to the mean; they were the most “typical” results. The average information provided by each neuron was the difference between *S*_*inf*_ for total entropy and the average *S*_*inf*_ for noise entropy, in other words, the distance in each case between the open and solid ×. For the spiking and bursting neuron, this worked out to be 0.16 and 0.53 bits/spike, respectively. (**B**) Information transmission for the spiking and bursting neuron for the experiments shown in A and for five other different combinations of the signal and noise filter frequencies. The amplitudes of the dc and time-varying components in each case were the same as in A. For each of the new combinations, the total entropy was calculated from 60,000 s of data, and five different 1-s duration signals (20,000–50,000 trials for each) were used to compute the noise entropy. Error bars correspond to standard deviations of the noise entropies

Figure 10A shows the results of this calculation. The stimuli used consisted of components with amplitudes and frequencies as in Fig. 9, and the *S*_*i*_ were computed as before. For the total entropy calculation, a long, non-repeating stimulus was used. In Fig. 10, the *S*_*i*_ (total entropy) that met the 2% criterion are plotted versus inverse dimension for the spiking (open circles) and bursting neuron (open squares), and linear fits were used to find *S*_*inf*_ (open ×) for each neuron. For the noise entropy calculation, the stimuli were repeats of a 1-s duration, 3 Hz signal to which different 1000 Hz waveforms were added during each trial. The results for two particular 3 Hz signals (different signals for the spiking and the bursting neuron) are shown on the graph: the *S*_*i*_ are plotted with solid circles (spiking) and solid squares (bursting) to which linear fits were made. The *S*_*inf*_ for these two signals are not indicated on the axis. The values of *S*_*inf*_ for the noise entropy varied with the 3 Hz signal that was used, and calculations were made for ten different randomly-generated 3 Hz signals for each neuron. The average *S*_*inf*_ for the spiking and bursting neurons are what are indicated by a solid × on the vertical axis; the error bars above and below indicate ± one standard deviation. (The *S*_*inf*_ for the two particular signals shown were close to the average values.) The average information transmitted, which is the difference between *S*_*inf*_ for total entropy and average *S*_*inf*_ for noise entropy, was 0.16 bits/spike for the spiking neuron and 0.53 bits/spike for the bursting neuron. Figure 10B replots the bursting and spike neuron information results of Fig. 10A along with those for five other combinations of signal and noise frequencies. (The amplitudes of the dc and time-varying components were the same as for Fig. 10A, and again 1-s duration stimuli were used to calculate the noise entropy.) Neither neuron transmitted much information when the noise was filtered at 100 Hz, near the peak in entropy for both neurons (Fig. 8). The bursting neuron transmitted more information than the spiking neuron for all signal frequencies when the noise filter frequency was 1000 Hz, although the spiking neuron did nearly as well as the bursting neuron when the signal frequency was filtered at 10 Hz, and *S*_10Hz_ / *S*_1000Hz_ had increased to 1.16 for the spiking neuron.

## Discussion

Bursts of spikes are observed in many sensory systems (Krahe & Gabbiani, 2004) and can be superior to individual spikes for encoding certain stimulus features. For example, in the electrosensory organ of the electric fish *Eigenmannia*, electroreceptor afferents describe the amplitude of a time-varying electric field in spike trains, while bursts produced by their targets, pyramidal cells, are a more reliable indicator of temporal features in the signal (Gabbiani et al., 1996). In spike trains produced by pyramidal cells in another electric fish, *Apteronotus leptorhynchus*, bursts signal a behaviorally-relevant, low-frequency feature in the stimulus, while isolated spikes describe the waveform of the entirety of the stimulus over time (Oswald et al., 2004). Ganglion cells in the salamander retina also produce both spikes and bursts, but the bursts have been observed to occur in response to a more specific distribution of stimuli (Smirnakis et al., 1997). In the primate lateral geniculate nuclei (LGN) and primary visual cortex (V1), bursts, rather than single spikes, were found to be produced more often after microsaccades, suggesting that bursts are a more reliable indicator of stimulus visibility (Martinez-Conde et al., 2002). An important distinction between our study and many of those that we reference is that the circle/circle neuron does not burst necessarily in response to a particular stimulus feature, but rather becomes increasingly sensitive to depolarizing fluctuations in the stimulus as the slow potassium conductance (responsible for ending the previous burst) decreases.

Just because a neuron efficiently transmits information about a stimulus does not mean that all features are well represented in the output or, conversely, that the stimulus can be accurately deduced from the spike train. This is clear in Fig. 3A, C: the slowly-filtered (0.1 Hz) stimulus clearly can be more readily reconstructed from the burst train than can the fast (1000 Hz) one, and yet the information capacity is much larger in the second case. A lower bound on the information can be computed using a reconstruction of the stimulus made from the spike train (Rieke et al., 1997). Reinagel et al. (1999) used this approach to measure the information transmitted by spikes and bursts (2–5 spikes) produced by thalamic relay cells in the cat dorsal LGN in response to a visual stimulus produced from white noise low-pass filtered at ~ 16 Hz. When they separated the spike train into one consisting of bursts (with any number of spikes) and the other of the spikes that were not in bursts, they found that the burst and tonic firing modes relayed roughly equivalent amounts of information (~ 2.5 bits/spike, using 4.96 ms bins) but that the coding efficiency was 1.5 times higher for the bursts, and three times higher if they were considered as unitary events.

The times at which spikes produced by a bursting neuron occur are inherently correlated, and these correlations can define patterns of spiking that encode stimulus features (Eyherabide et al., 2009). Reich et al. (2000) showed that, in primate V1, information conveyed by a spike depended on the duration of the previous ISI and that, similar to the observations of Reinagel et al. (1999), short-ISI spikes associated with doublets (two-spike bursts) were particularly important for information transmission. In the *Apteronotus* pyramidal cells mentioned above, the ISIs within a burst were observed to encode stimulus intensity (Oswald et al., 2007). Moreover, real bursts were found to transmit more information than spike trains consisting of only the first spikes of the bursts, while artificial burst trains, in which stochastically-timed spikes were added back to the same spike trains, transmitted less (Schlungbaum et al., 2025). Two other studies of note have considered coarse features of bursts when computing information. Eyherabide et al. (2008, 2009) used a correlation-based approach to identify *n*-bursts (bursts consisting of *n* spikes) in spike trains recorded intracellularly from axons of grasshopper auditory receptors in animals exposed to a stimulus that was white up to one of five cutoff frequencies that varied from 25 to 800 Hz. Over all experiments, the average information transmitted was 1.7 ± 0.6 bits/spike (or 2.0 ± 0.6 bit/spike using the direct method). Kepecs and Lisman (2003, 2004), using a two-compartment model neuron with a random current stimulus (5 Hz cutoff frequency) to which white noise was added, concluded that duration was the most important property of a burst, beyond its start time, when calculating information. Most pertinent to our own investigation in these two studies were the observations that representing the burst only by its first spike preserved 74% and 70% of the information rate, respectively. When spikes in a burst (Eyherabide et al., 2009) or burst duration (Kepecs & Lisman, 2003) was also considered, the numbers rose to 94% and 92%. In our simulation using a 10 Hz signal (Fig. 6B), the corresponding values were 54% of the total entropy when using only the first spike in a burst and 87% when also considering the last one. Considering the period and number of spikes in a burst, instead, only accounted for 66% of the entropy.

Entropy (i.e. information capacity), rather than information, was the primary focus of this work, and, as in other investigations, most of our calculations treated bursts as unitary events, characterized by their start times. The peak in the entropy for filtering at 30 Hz was not surprising. The periods of consecutive bursts were uncorrelated and, on average, the neuron was seeing three or four wiggles in the stimulus in the time since the preceding burst, providing more variability in the response than that for a 10 Hz signal, for which each upturn typically triggered a burst (Fig. 3B). A result that was more surprising to us was the steep jump in entropy/burst between 1 and 3 Hz (Fig. 5), corresponding to the transition from highly-correlated burst periods (and something akin to a rate code) to independent ones. The increase itself was not unexpected, but, a priori, we would not have predicted the sharpness or the location of this feature or the similar transition in the spiking model neuron. This observation motivated our choice of a 3 Hz signal and 1000 Hz for the information calculations in which we found that the bursting neuron transmitted more than three times as much information than the spiking neuron and had ten times the coding efficiency (Fig. 10). Another curious result was that, although the timing of the starts of consecutive bursts was uncorrelated for intermediate filter frequencies (3–30 Hz), correlations returned at higher frequencies (100–1000 Hz) that the neuron largely ignored. This resulted from the period being dependent on the number of spikes in the previous burst (Fig. 3C).

Filtered white noise is not necessarily a physiologically-relevant stimulus, but it is often used in both in both experimental and computational investigations, including the ones discussed in this paper. The GPR2 neuron that inspired our theoretical investigation is unlikely to be subjected to a filtered white-noise stimulus in vivo but most likely is stimulated quasi-periodically by muscle contractions during the rhythmic gastric mill motor pattern, whose frequency (0.05–0.2 Hz) (Coleman et al., 1995; Mulloney & Selverston, 1974; Selverston and Mulloney, 1974; Weimann et al., 1991) is comparable to the intrinsic bursting frequency (0.01–0.05 Hz) of the neuron measured in unstretched neuromuscular preparations in vitro (Birmingham et al., 1999). It would be interesting to revisit coding in the GPR2 system and to measure the entropy of (and possibly the information transmitted by) burst trains produced in response to white-noise stimuli low-pass filtered at physiologically-relevant frequencies by considering burst durations and IBIs.

Accurate spike train entropy and information calculations require that the associated probabilities be estimated accurately. Most of the results presented in this paper could not have been obtained using experimental data; some of our simulations analyzed mathematical burst trains that were more than a hundred hours in duration, longer than any possible lab experiment and even longer than the time over which one could expect a living system to remain in a stationary state with constant maximal conductances.

The most notable conclusion of this study is that the circle/circle bursting neuron is capable of transmitting more information than might be expected, and that its response is particularly robust when the signal (but not the noise) is well-matched to the model’s slow dynamics. It would be interesting to repeat these simulations while varying the dc and rms amplitudes of the stimulus, which would affect the average burst period and the fraction of a period over which fluctuations could trigger bursts, respectively. 

The circle/circle neuron is just one of many different mathematical bursting neurons that can be categorized using the tools of dynamical systems theory (Izhikevich, 2000). One feature of this model is that Eqs. 3 and 4, which govern the dynamics of the two slow conductances, are uncoupled. Bursting is not autonomous but rather requires feedback from the membrane potential *V* and hence depends on the activity of the spike-producing conductances (Izhikevich, 2006). Whether this interplay has a role in information transmission and whether our results will generalize to other models of endogenously-bursting neurons merit further analysis.

## Data Availability

Available upon request.
